# Visceral leishmaniasis with cardiac involvement in a dog: a case report

**DOI:** 10.1186/1751-0147-51-20

**Published:** 2009-04-30

**Authors:** Mónica López-Peña, Nuria Alemañ, Fernando Muñoz, Dolors Fondevila, María Luisa Suárez, Ana Goicoa, Jose María Nieto

**Affiliations:** 1 Hospital Clínico Veterinario Rof Codina, Dpto. Ciencias Clínicas Veterinarias, Universidade de Santiago de Compostela, Spain; 2 Dpto. de Anatomía y Producción Animal, Facultade de Veterinaria de Lugo, Universidade de Santiago de Compostela, Spain; 3 Dpt. Medicina i Cirurgia Animals, Facultat de Veterinària, Universitat Autònoma de Barcelona, Spain

## Abstract

A dog presented with cutaneous nodules, enlarged lymph nodes and oedema in limbs, face and abdomen. The diagnosis of visceral leishmaniasis was established by identification of *Leishmania *amastigotes within macrophages from skin and popliteal lymph node biopsies. At necropsy, lesions were found in different organs, but it was particularly striking to observe large areas of pallor in the myocardium. Histological examination revealed an intense chronic inflammatory reaction in many organs, and numerous macrophages were found to contain amastigote forms of *Leishmania*. The inflammatory reaction was especially severe in the heart, where large areas of the myocardium appeared infiltrated with huge numbers of mononuclear immune cells, causing cardiac muscle atrophy and degeneration. Despite the severe inflammation, the number of parasitized macrophages was low in the myocardium, as revealed by immunohistochemical staining of *Leishmania *amastigotes. Because cardiac involvement is not usually described in this condition, this dog represents a very rare case of canine visceral leishmaniasis with affection of the myocardium.

## Background

Canine leishmaniasis is a zoonotic parasitic disease caused by an intracellular protozoan of the genus *Leishmania *that is transmitted to the dog by the bite of blood-sucking sand flies (*Phlebotomus *species). The parasite reproduces itself within the host's macrophages and, as a consequence, causes an intense mononuclear inflammatory reaction consisting of mononuclear immune cells. In the Mediterranean countries, *L. infantum *is the species usually implicated in canine visceral leishmaniasis, a severe systemic form of the disease characterized by progressive wasting due to the involvement of multiple organs such as spleen, liver, lymph nodes, bone marrow, kidneys and skin [1,2]. The clinical presentation of canine visceral leishmaniasis vary widely depending both on the organ(s) affected and the extent of functional impairment caused by the infection, and therefore, diagnosis may become a real challenge, particularly if the animal do not live in an endemic area or, alternatively, if the disease appears in an unusual clinical form [3]. In this report we describe an atypical form of canine visceral leishmaniasis with severe affection of the myocardium.

## Case presentation

An 11-year-old female Basset Hound was presented to the Hospital Clinico Veterinario Rof Codina with a history of swelling of the head and limbs. On initial evaluation, abnormal physical examination findings included non-ulcerated cutaneous nodules, a generalized mild lymphadenomegaly of the superficial lymph nodes, a stiff gait and oedema in limbs, face and abdomen. Haematological abnormalities included normochromic anaemia (haematocrit value of 32.9 per cent), lymphopenia/monocytopenia (0.6 × 10^9^/l), thrombocytopenia (54 × 10^9^/l) and hypoalbuminemia (2.10 g/dl). Blood urea nitrogen (BUN) and creatinine values were under normal ranges, but urine specific gravity was 1.025, with protein level of 2+. An electrocardiogram (ECG) showed low amplitude of the QRS complex with normal cardiac rate and rhythm. Routine histological evaluation of biopsies obtained from the skin and the popliteal lymph node revealed a chronic non-suppurative inflammation consisting mainly of macrophages and some plasma cells and lymphocytes. Small vacuoles containing basophilic bodies morphologically consistent with *Leishmania *amastigotes were also seen in the cytoplasm of a few macrophages and therefore, visceral leishmaniasis was diagnosed.

A specific treatment was proposed for the dog by the practitioner, but refused by the owner. At the owner's request, the dog was euthanized two days later.

At post-mortem examination, the main abnormal external findings were multiple cutaneous nodules and generalized enlargement of the superficial lymph nodes as already noticed clinically. Abdominal lesions included splenomegaly and fibrotic, cortical striation and the presence of small whitish nodular foci in the renal cortex. The lungs were mottled tan. Multifocal areas of pallor affected the left ventricular and septal myocardium.

Samples of all organs were fixed in 10% neutral buffered formalin and embedded in paraffin according to standard laboratory procedures. Paraffin sections, 5 μm-thick, were stained with hematoxylin and eosin (H-E) and Giemsa stains, and evaluated by light microscopic examination. The presence of *Leishmania *amastigotes in tissues was investigated by indirect immunoperoxidase staining by using a rabbit polyclonal anti-*Leishmania *antibody [4].

**Figure 1:**
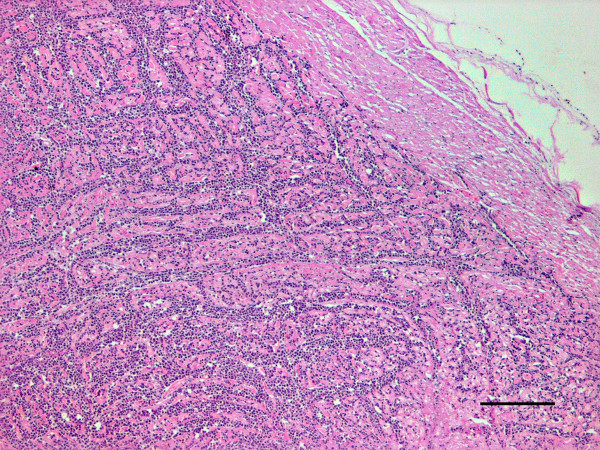
**Granulomatous myocarditis**. Severe interstitial infiltration of macrophages and some lymphocytes and plasma cells in the myocardium. HE. Bar = 200 μm.

**Figure 2:**
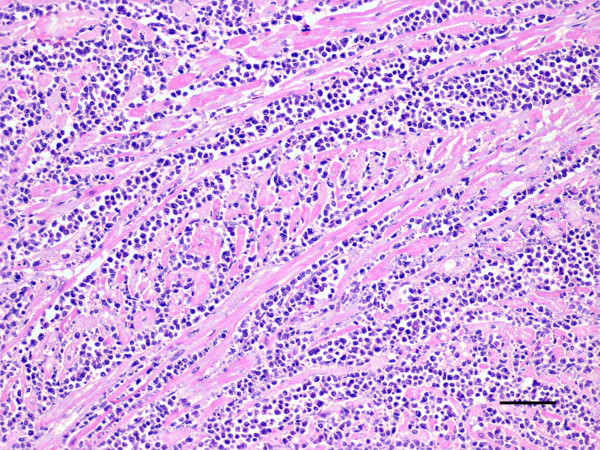
**Granulomatous myocarditis**. Non-suppurative myocarditis with cardiac muscle atrophy, degeneration and loss of myocardiocytes. HE. Bar = 200 μm.

The most important histopathological finding was the presence of an intense chronic inflammatory reaction composed of mononuclear cells (predominantly monomorphic macrophages, but also plasma cells and lymphocytes) in most organs. In the heart, the myocardium showed a dense accumulation of macrophages between the muscle fibers (Fig 1) and also areas of cardiac muscle atrophy, degeneration and loss of myocardiocytes (Fig 2). The lungs showed small foci of interstitial thickening associated with bronchioles and small vessels; these foci displayed mononuclear infiltration, which were also observed in the pleura. In the liver, small aggregates of large macrophages were found throughout the parenchyma and, in the kidneys, we found evidence of membranoproliferative glomerulonephritis, as well as of interstitial infiltration of inflammatory mononuclear cells. The spleen exhibited a marked lymphocyte depletion affecting mainly the periarterial lymphatic sheaths; in agreement with the clinical finding of anaemia, large numbers of single megakaryocytes were observed in the red pulp.

The parasite load was high in spleen, lymph nodes and liver, organs where most of the macrophages had large cytoplasmic parasitophorous vacuoles with round basophilic nucleus and distinct bar-shaped paranuclear kinetoplast, morphological features compatible with *Leishmania *amastigotes. In lungs, kidneys and skin, amastigote forms of *Leishmania *were seen inside macrophages, but with restricted distribution in the inflammatory infiltrate. Notably, the parasite load was extremely low in the heart, since only a few macrophages were parasitized, requiring careful search to identify them in a background of severe inflammation.

**Figure 3:**
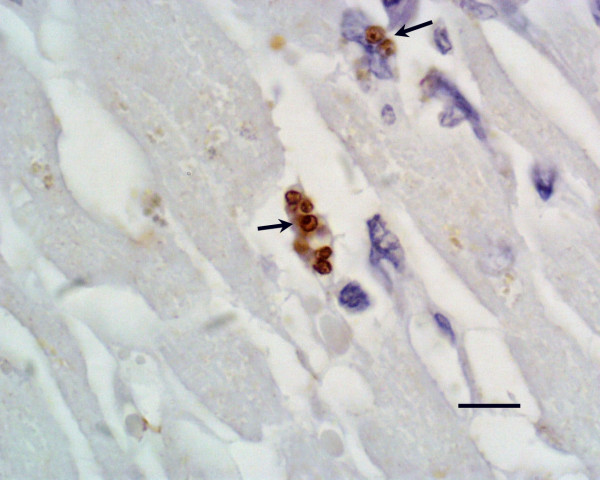
***Leishmania *in myocardium**. Amastigotes of *Leishmania *(arrows) inside macrophages located between muscular fibers of the myocardium. Anti-*Leishmania *antibody, indirect immunoperoxidase staining, mild hematoxylin counterstain. Bar = 6 μm.

The identification of parasitized macrophages was greatly facilitated in all organs, but especially in the heart, by the immunohistochemical detection of *Leishmania *antigens, resulting in a strong labelling of amastigotes inside macrophages (Fig 3).

## Conclusion

Cardiac involvement in canine visceral leishmaniasis appears to be anecdotal, since only a very few cases of myocardiopathy during the course of the disease have been reported in the literature to date [5-7]. However, only in a recent study, to the authors' knowledge, a direct relationship between the presence of lesions in the myocardium and *Leishmania *amastigotes has been undoubtedly demonstrated [8].

In the case we report here, the occurrence of subcutaneous oedemas was attributed to hypoalbuminemia as a result of nephropathy with protein loss, with changes in the ECG that correlated with this initial diagnosis. After the definitive diagnosis of visceral leishmaniasis, renal dysfunction can be explained by glomerulonephritis due to deposition of immune complexes, while changes in ECG may be also explained by the occurrence of myocarditis caused by *Leishmania*. Therefore, affection of the myocardium and, as a consequence, impairment of the cardiac function must be added to the plethora of lesions and clinical signs that may be present in dogs with the visceral form of this chronic parasitic disease.

## Competing interests

The authors declare that they have no competing interests.

## Authors' contributions

MLP and JMN were responsible for the necropsy and the histological examination. NA and DF were responsible for the immunohistochemistry. FM, AG and MLS were responsible for the clinical examination of the dog. All authors were involved in drafting the manuscript and gave final approval of the manuscript.
